# Reinforcement Learning-Based Management in IoT-Enabled Renewable Energy Communities: An Approach to Optimization for Comfort, Economy, and Sustainable Performance

**DOI:** 10.3390/s26051682

**Published:** 2026-03-06

**Authors:** Stefano Caputo, Eleonora Iacobelli, Maurizio De Lucia, Sara Jayousi, Lorenzo Mucchi

**Affiliations:** 1 Department of Information Engineering, University of Florence, 50139 Florence, Italy; lorenzo.mucchi@unifi.it; 2 Department of Industrial Engineering, University of Florence, 50139 Florence, Italymaurizio.delucia@unifi.it (M.D.L.); 3 PIN Foundation, Prato Campus of University of Florence, 59100 Prato, Italy; sara.jayousi@pin.unifi.it

**Keywords:** Renewable Energy Communities, Internet of Things, reinforcement learning, energy management, smart grid

## Abstract

The increasing deployment of Internet of Things (IoT) sensing infrastructures and distributed renewable energy resources is enabling the emergence of Renewable Energy Communities (RECs), which require intelligent, adaptive, and decentralized energy management strategies. This study proposes a sensor-driven reinforcement learning (RL) framework for the coordinated management of residential RECs, aiming to jointly optimize thermal comfort, economic savings, and environmental sustainability. Each household is equipped with a network of IoT sensors monitoring indoor temperature, energy production and consumption, battery state of charge, and user presence, which collectively define a discretized state space for a tabular Q-learning agent controlling heating systems and programmable appliances. A stochastic simulation environment is developed to realistically reproduce weather variability, building thermal dynamics, user activity profiles, and photovoltaic generation. To address the instability typical of multi-agent learning, a two-stage training strategy is adopted: agents are first pre-trained at single-house level using synthetic sensor data and are subsequently deployed within the full community, where coordination is achieved through shared reward components without explicit inter-agent communication. Performance is evaluated on a heterogeneous Renewable Energy Community (REC) composed of eleven households, including both prosumers and consumers. The simulation results show that the proposed approach significantly outperforms rule-based control strategies, achieving lower energy consumption, improved thermal comfort stability, and higher global reward. Moreover, pre-trained agents maintain stable and cooperative behavior when operating concurrently at community level, with limited sensitivity to exploration. These findings demonstrate that sensor-driven, lightweight reinforcement learning represents a viable and scalable solution for decentralized energy management in IoT-enabled Renewable Energy Communities.

## 1. Introduction

Recent advances in artificial intelligence have enhanced the autonomy and adaptability of Internet of Things (IoT) and Wireless Sensor Networks (WSNs). Among various methods, learning-based approaches have gained particular attention for their ability to optimize control strategies in dynamic and uncertain environments. Unlike traditional approaches based on predefined rules, these agents learn through continuous interaction with the environment, adjusting their behavior to maximize performance over time. When applied to energy management, this paradigm enables efficient scheduling, resource allocation, and real-time decision-making in smart grids and microgrids [1]. The integration of Reinforcement Learning (RL) with IoT-based sensing and communication allows distributed nodes to act intelligently, reducing dependence on centralized computation and improving responsiveness in energy systems [2].

Renewable Energy Communities (RECs) represent a decentralized model for local energy production and sharing, where citizens, companies, and institutions jointly manage renewable resources. Their emergence reflects a shift from centralized energy systems toward community-based structures that promote sustainability, resilience, and social participation [3]. Within these communities, the integration of multiple renewable sources, such as solar, wind, and storage systems, creates a complex ecosystem that requires smart management to balance variable generation and fluctuating demand [4]. IoT devices and WSNs enable fine-grained monitoring of distributed assets, providing real-time data that can support predictive and adaptive management at both the household and community levels [5].

However, the stochastic nature of renewable energy and the heterogeneity of consumption profiles introduce significant operational challenges. Variability in solar irradiation, wind speed, and load patterns affects the stability and efficiency of community energy exchanges, demanding advanced control strategies capable of handling uncertainty and dynamic conditions [6]. To achieve sustainable and economically viable operation, RECs increasingly rely on AI-driven frameworks capable of coordinating decentralized agents, forecasting renewable generation, and scheduling flexible loads in real time [7]. These intelligent control systems enhance community self-consumption and cost efficiency while reducing dependence on the external grid, contributing to broader decarbonization goals.

The development of RECs has been strongly driven by recent European legislation promoting citizen participation in the energy transition. The recast Renewable Energy Directive (EU) 2018/2001 establishes a legal framework for collective self-consumption and empowers local communities to produce, share, and trade renewable energy within a common infrastructure [8]. Furthermore, Directive (EU) 2019/944 on common rules for the internal electricity market defines the rights and responsibilities of active consumers and energy communities, emphasizing fair access to the grid and the integration of distributed generation [9]. These regulatory initiatives provide the foundation for implementing intelligent, data-driven management systems that ensure economic fairness, transparency, and sustainability in community-based energy ecosystems.

Although RECs offer a promising framework for local decarbonization, their operation remains highly complex due to the variability of renewable resources and the stochastic nature of user behavior. The interplay between distributed generation, storage, and flexible demand results in a multi-objective optimization problem involving comfort, cost, and sustainability. Classical optimization approaches often assume deterministic models or rely on static parameters, which limits their applicability in dynamic contexts characterized by uncertainty and incomplete information [10]. Moreover, rule-based control strategies typically require centralized supervision, leading to scalability issues and delayed responses in large-scale community networks [11].

To overcome these limitations, recent research has focused on data-driven and RL techniques capable of autonomously adapting to system dynamics [12]. When integrated with IoT and WSN infrastructures, RL-based agents can exploit real-time sensor data to optimize load scheduling, energy sharing, and resource allocation, enhancing both efficiency and user comfort. This combination of intelligent sensing and adaptive control represents a key step toward fully self-organizing and resilient RECs [13].

Despite the growing interest in intelligent management of RECs, several challenges remain unsolved. The system state in energy communities is defined by multiple continuous variables (e.g., sensor measurements, power flows, storage levels), which require discretization to be modeled within a Markov Decision Process framework (see Section 2.2). The combinatorial growth of the resulting discrete state space, together with the heterogeneity of household behavioral profiles (e.g., occupancy schedules and consumption patterns), makes it challenging to design control strategies that generalize across different contexts. Furthermore, the coupling between stochastic renewable generation and dynamic pricing mechanisms introduces non-stationarity into the learning environment, often leading to unstable convergence of RL agents [14]. In addition, most existing frameworks neglect the coordination among multiple agents, resulting in suboptimal energy sharing and unbalanced incentives [15]. Addressing these issues requires the development of distributed and stable learning architectures that can balance exploration and exploitation while ensuring fairness and economic viability within the community [16].

In this work, we propose a collaborative multi-agent reinforcement learning framework tailored to the management of RECs. The learning environment is modeled as a stochastic system, where statistical data and Gaussian noise introduce non-deterministic dynamics that more closely reflect real-world operating conditions. Collaboration among agents is facilitated through an external computation module responsible for aggregating community-level information and deriving part of the reward signals. Specifically, this module collects operational data from the Renewable Energy Community (REC) and provides two out of the three reward components required for agent training, thereby ensuring coordinated and consistent learning across the network. Each agent is implemented using a tabular Q-learning algorithm, which maps every possible input state to an action determined by its associated Q-value. This design enables agents to autonomously adapt their decisions in response to fluctuating renewable generation and demand, while maintaining a cooperative strategy that enhances overall community performance. By combining distributed sensing, stochastic modeling, and collaborative learning, our approach aims to achieve resilient, scalable, and economically viable energy management within RECs. Although Reinforcement Learning approaches are known to suffer from relatively long convergence times and high computational requirements, the proposed method is designed with the objective of limiting the training time. This aspect is particularly relevant in energy communities, where changes such as variations in household occupancy, user profiles, or demand patterns may require periodic retraining of the control policy. Therefore, achieving a reasonably fast convergence is essential to ensure adaptability of the system in dynamic real-world scenarios.

### 1.1. Related Works

Recent research has explored a variety of strategies to manage and optimize RECs, reflecting the complexity and heterogeneity of distributed energy systems. Approaches in the literature can be broadly grouped into three clusters: traditional centralized optimization, learning-based centralized methods, and multi-agent decentralized strategies. Moreover, hybrid solutions emerge at the intersections of these clusters, combining different methodologies to exploit their respective strengths.

The *Optimization-based approach* focuses on centralized strategies that gather system-wide data and optimize REC operations using classical optimization techniques. Mixed-integer linear programming (MILP) has been widely applied for energy scheduling and coordination within RECs [4]. Giordano et al. [2] propose a central optimization framework for IoT-aware energy exchanges using rule-based and optimization techniques. Dal Cin et al. [17] explore multi-criteria optimization to balance economic and environmental objectives, while Ayodele et al. [18] present rule-based load management strategies for PV/battery-powered residential buildings.

The *Learning-based approach* leverages reinforcement learning (RL) and deep learning techniques in a centralized setup. Song et al. [12] use deep neural networks for flexible load disaggregation, whereas Alam et al. [19] propose a deep learning-based control for community energy storage. Sebastianelli et al. [20] investigate deep Q-learning applications for control tasks, highlighting the potential of RL in energy management, while Franz et al. [14] discuss instabilities in deep Q-learning models, emphasizing the challenges of learned-based approaches in dynamic environments.

The *Multi-agent approach* considers decentralized architectures where agents exchange information at a high level without fully centralizing the optimization process. Zhou et al. [15] present a peer-to-peer energy trading framework, and Bilardo [21] develops fairness-aware multi-agent incentive mechanisms. Brusco et al. [22] combine distributed storage optimization with a multi-agent structure to enable efficient energy sharing across the community.

Hybrid approaches address the intersections between clusters. Solutions combining traditional optimization with learning-based methods in a centralized manner are reported by Ponnaganti et al. [7], who integrate pre-processed data with RL-based flexibility management. The intersection of centralized optimization and decentralized architectures is explored by Koirala et al. [3], who implement decentralized community energy storage using classical optimization. Fully decentralized RL or learning-based strategies are exemplified by Friess et al. [13], employing forecast-based multi-agent RL optimization. Finally, Di Somma et al. [5] present a decentralized hybrid approach that combines optimization and AI-based methods, addressing both operational efficiency and incentive maximization.

These studies collectively demonstrate the variety of methods applied to REC management, from fully centralized optimization to decentralized learning-based architectures. The hybridization of approaches shows particular promise, especially for large-scale communities where scalability, adaptability, and fairness must be balanced. This survey provides a foundation for positioning our proposed methodology within the landscape of REC optimization and control, highlighting the rationale for adopting a multi-agent RL framework in a decentralized setting.

In summary, the existing literature highlights a clear trade-off between performance and scalability. Centralized approaches, whether optimization-based or learning-based, generally achieve high control quality but rely on extensive data aggregation and large state–action spaces, which limits their applicability in real Renewable Energy Communities composed of hundreds of dwellings. Conversely, decentralized and multi-agent strategies offer better scalability by relying only on local information, yet most studies do not fully address how locally trained agents can be prepared to operate effectively within a cooperative community. Motivated by these limitations, the next section presents our contributions, which introduce a pre-training strategy for single agents, a simulation-based method to emulate community interactions during learning, and a full validation of the resulting policies within a multi-agent REC environment simulation.

### 1.2. Our Contributions

This study addresses the complexity of managing REC by combining simulation, parametric modeling, and reinforcement learning techniques. The proposed framework has been designed not only to evaluate the potential benefits for individual participants, but also to provide scalable and adaptable solutions that reflect the heterogeneity of community structures. In addition, particular attention has been devoted to the efficiency of agent training and to the possibility of leveraging synthetic data for pre-training, thereby facilitating practical deployment in real-world scenarios. The contributions of this work can be summarized as follows.

- Simulation tool for REC evaluation: A simulation framework has been developed to quantify the benefits for individual users within a REC. This tool enables systematic assessment of economic and operational advantages of community-based energy sharing.
- Parametric and scalable architecture: A parametric design has been introduced to ensure scalability and adaptability to different REC configurations. By adjusting system parameters, the framework can be tailored to the specific needs and consumption profiles of each user, supporting heterogeneous and evolving community structures.
- Fast and independent training algorithm: A tabular Q-learning algorithm has been implemented, which can be trained rapidly and independently for each agent. In contrast to approaches requiring continuous inter-agent communication, this method allows local training while maintaining cooperative performance, thereby reducing computational overhead and communication costs.
- Synthetic data generation for pre-training: The simulation framework can also serve as a generator of synthetic datasets, which may be employed to pre-train agents. This capability reduces the training time required in real-world environments, where energy performance is often inefficient, and facilitates more practical deployment of reinforcement learning in RECs.

These contributions establish a flexible and efficient foundation for intelligent REC management, combining scalability, adaptability, and the potential for accelerated real-world implementation.

The remainder of this paper is organized as follows. Section 2 introduces the simulation framework, including the external inputs, the reinforcement learning agent, and the residential environment model. Section 3 presents the reward formulation guiding the learning process. Section 4 reports the experimental outcomes from both the single-house pre-training and the full Renewable Energy Community simulation. Finally, Section 5 provides a critical analysis of the results, emphasizing their importance for community-level energy management, and Section 6 summarizes the main findings and outlines future research directions.

## 2. Materials and Methods

RECs are autonomous entities that unite citizens, small businesses, and local authorities to collaboratively produce, consume, store, and share renewable energy. Their purpose is not profit generation but the delivery of environmental, economic, and social benefits, fostering cooperation, local empowerment, and energy democracy. Participants may act as prosumers (i.e., entities that simultaneously produce and consume energy), pure consumers, or pure producers, with voluntary membership motivated by independence, cost savings, and sustainability goals. RECs decentralize energy production, promote renewable technologies, and strengthen community resilience, while IoT devices and Wireless Sensor Networks provide real-time monitoring of generation, storage, and consumption. These data streams enable predictive and adaptive control strategies, allowing communities to balance variable supply with fluctuating demand, optimize self-consumption, and reduce reliance on the external grid.

In modern RECs, sensing and actuation technologies play a central role in enabling intelligent and adaptive energy management. Each dwelling typically integrates a set of IoT sensors that monitor key physical variables, such as indoor air temperature, domestic hot water temperature, and appliance usage, which are essential for assessing occupant comfort and modeling thermal dynamics. Additional sensors measure photovoltaic production, battery state of charge, and real-time electricity consumption, providing the feedback required to evaluate energy balance and to support optimization strategies aimed at reducing costs and improving self-consumption. On the actuation side, home automation systems allow direct control of thermostats, heat pumps, air conditioners, and major household appliances, enabling demand shifting and the coordinated use of flexible loads. These devices act as controllable energy assets that can be scheduled or modulated according to renewable availability, thereby enhancing the responsiveness and efficiency of the community-level control framework.

**Figure 1:**
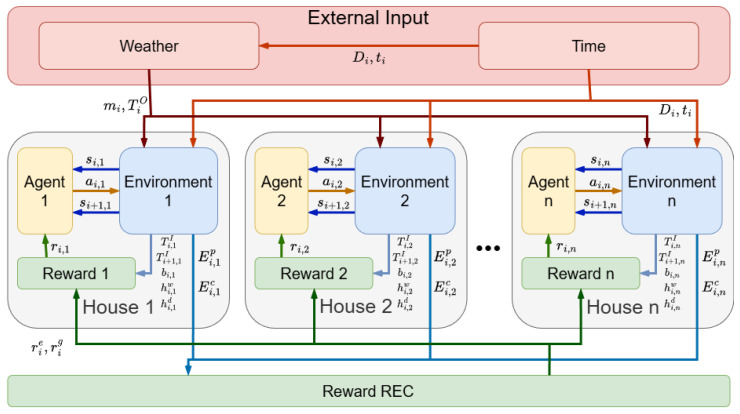
System Model.

Figure 1 illustrates the architecture of the proposed simulation framework, which models the operation of a Renewable Energy Community through a multi-agent reinforcement learning system. The framework is structured around a set of residential units, each equipped with an agent and a corresponding environment.

The system receives four external input variables: $D_{i}$ (day of the year), $t_{i}$ (time slot of the day), $T_{i}^{O}$ (external temperature), and $m_{i}$ (external weather condition). The latter two variables are derived through statistical analysis based on the values of $D_{i}$ and $t_{i}$, introducing stochasticity into the simulation. These external inputs represent environmental conditions surrounding the community and influence the behavior of each agent’s environment in a non-deterministic manner.

Based on these inputs and additional internal variables each environment generates an initial state $s_{i,n}$ at time step *i* for the *n*-th house. This state is processed by the corresponding agent to produce an action $a_{i,n}$, which controls certain smart devices within the environment. The action modifies the environment, resulting in a new state $s_{i+1,n}$ at the next time step.

Several parameters measurable by sensors, such as the indoor temperature ($T_{i,n}^{I}$), contribute to the computation of the “Thermal and Service Comfort” reward ($r_{i,n}^{c}$), which is specific to each individual house (in figure it indicated as Reward 1,2, …, n). Other measurable parameters, such as the energy produced $E_{i,n}^{p}$ and consumed $E_{i,n}^{c}$, are used by a centralized computation module to determine the community-level rewards: Economic Saving ($r_{i,n}^{e}$) and Green Sustainability ($r_{i,n}^{g}$).

The final reward $r_{i,n}$ used to update the Q-value for each agent is computed as the average of the individual comfort reward and the two community-level rewards. This mechanism ensures that each agent learns to optimize both personal comfort and collective performance within the REC. All rewards described in detail in Section 3.

Although the overall REC environment is currently simulated, the underlying appliance, occupancy, and PV production models are calibrated and validated with real-world measurements, ensuring that the simulation reflects realistic patterns of energy generation and consumption.

### 2.1. External Input

The simulation framework is based on a discrete temporal structure, where each step is represented by the variable $t_{i}$. This variable indicates the daily time slot, with the day divided into eight slots of equal duration. Each slot corresponds to a 3 h interval, such that a complete day is covered by eight consecutive steps. At the end of the eighth slot, $t_{i}$ resets to 0 and the day index $D_{i}$ is incremented.

The variable $D_{i}$ represents the day of the year and can assume integer values in the range [0,364]. Once the annual cycle is completed, $D_{i}$ also resets to 0, allowing the simulation to proceed over multiple years. The total number of simulated years depends on the specific training or testing configuration adopted.

This discretization of time enables the modeling of both daily and seasonal dynamics, ensuring that the agents are exposed to recurrent patterns of variability in environmental conditions and user behavior. The combination of $t_{i}$ and $D_{i}$ thus provides the temporal backbone of the simulation, supporting the generation of external inputs and the evolution of the system states across different time horizons.

The weather block provides the environmental boundary conditions for the simulation of energy balance in each household. It generates two main variables: the discrete meteorological condition $m_{i}$ and the outdoor temperature $T_{i}^{0}$, updated at each simulation time step $t_{i}$. The model combines empirical weather statistics with stochastic sampling to reproduce realistic climatic variability over time.

The meteorological condition $m_{i}$ is selected from a discrete set of sky states (Clear, Mostly Clear, Partly Cloudy, Mostly Cloudy, Overcast) according to the probability of clear-sky occurrence for the corresponding day of year $D_{i}$. These probabilities were derived from monthly clear-day frequencies obtained from the Italian meteorological database 3bmeteo [23] and interpolated to daily resolution using a piecewise cubic Hermite polynomial. A uniform random draw determines the actual state, transforming continuous seasonal trends into discrete weather categories suitable for simulation. The occurrence of rainfall is introduced as a conditional event for all non-clear conditions, with monthly probabilities estimated from NASA MERRA-2 reanalysis data and historical records from Weather Spark [24]. This ensures seasonal and statistically consistent distributions of wet and dry days while excluding logically inconsistent states such as “Clear + Rain”.

However, the outdoor temperature $T_{i}^{0}$ is generated as a stochastic variable consistent with both seasonal and diurnal cycles. Monthly minimum, mean, and maximum temperature profiles were obtained from long-term observations (1980–2016) for the city of Perugia and adjusted using data from four nearby meteorological stations (Perugia LIRZ, Arezzo LIQB, Viterbo LIRV, and Grosseto LIRS), corrected for elevation and local bias according to the International Standard Atmosphere model. For each month, the simulation day is divided into characteristic time bands (night, morning, afternoon, and evening), and $T_{i}^{0}$ is sampled within realistic temperature intervals defined by the monthly minima and maxima. This approach captures intra-day temperature variability without introducing excessive randomness.

The resulting outputs $\left(m_{i},\;T_{i}^{0}\right)$ are provided to the thermal and electrical sub-models, influencing photovoltaic generation, heating and cooling demand, and overall comfort-driven control policies. The weather generator thus acts as a compact yet statistically grounded driver linking environmental variability to the energy management dynamics of the simulated renewable energy community. The overall statistical distribution of the generated data matches the observed seasonal averages, while the relatively high intra-day variance provides a conservative estimation of environmental variability, enabling robustness testing of the proposed control and optimization framework.

Although the present study adopts climate data from Perugia for model calibration and validation, the proposed simulation framework is not geographically constrained. Climate datasets (e.g., solar irradiance and temperature profiles) can be replaced with data from other regions without modifying the core structure of the model, enabling straightforward adaptation to different climatic scenarios.

### 2.2. Reinforcement Learning Agent: State and Actions

RL represents a class of machine learning techniques designed for sequential decision-making problems, in which an autonomous agent interacts with a dynamic or uncertain environment to learn a policy that maximizes the expected cumulative reward. The reward function encodes the objectives of the control problem and provides the agent with a quantitative measure of performance, thereby guiding the learning process [20]. Unlike supervised learning, RL does not rely on an external teacher to provide correct input–output pairs; instead, the agent improves its behavior through a reward–punishment mechanism that reinforces successful actions and penalizes suboptimal ones. By learning control strategies directly from experience, RL avoids the need for explicit system modeling or accurate forecasts of future states. This characteristic makes RL particularly suitable for real-time operational optimization in complex and uncertain environments, where traditional model-based approaches may become impractical or computationally prohibitive [25].

In this framework, RL problems are typically modeled as Markov Decision Processes (MDPs), a well-established mathematical formalism for sequential decision-making under uncertainty. An MDP is characterized by a state space *S*, which describes the relevant environmental conditions, such as internal and external temperatures in building applications, and an action space *A*, representing the set of control decisions available to the agent. The system dynamics are encoded in the transition probability function *P*, which specifies the likelihood of moving from one state to another after a given action. The reward function *R* quantifies the immediate benefit associated with each state–action pair and serves as the primary driver of the optimization process. The discount factor γ regulates the trade-off between short-term and long-term performance, while the initial state distribution $p_{0}$ defines the starting conditions of the decision process. Together, these elements provide a compact and expressive representation of the environment, enabling the agent to learn an optimal policy that maximizes long-term cumulative rewards.

Given the same initial state $s_{t}$, the agent may select different actions depending on its policy.

(1) π(·∣s):S→Δ(A)

defines a mapping from states to probability distributions over the entire action space A.

Among the various RL algorithms, this work adopts Q-learning, a model-free approach that estimates the expected value of taking a given action in a specific state through the function Q(s,a). In RL settings, each action affects both immediate and future outcomes, giving rise to the well-known trade-off between *exploration*, namely selecting actions that have not yet been fully assessed, and *exploitation*, i.e., leveraging current knowledge to maximize rewards. Q-learning addresses this challenge by iteratively updating the value of Q(s,a) based on the agent’s interactions with the environment, progressively refining its estimate of long-term returns for each state–action pair [26]. To regulate this balance, a widely used strategy is the ε-greedy action selection, in which the agent chooses the action with the highest estimated value with probability 1−ε, while with probability ε it selects a random action to encourage exploration. This mechanism ensures that the agent continues to improve its action-value estimates over time, reducing the risk of premature convergence to suboptimal policies and supporting a more robust learning process.

The Q-value function represents the expected cumulative reward obtained by executing action *a* in state *s* and subsequently following the learned policy. Its update rule is defined for the *n*-th house as:

(2) $$Q\left(s_{i,n},a_{i,n}\right)=Q\left(s_{i,n},a_{i,n}\right)+\alpha \left[r_{i+1,n}+\gamma \max_{a^{\prime }}Q\left(s_{i+1,n},a^{\prime }\right)-Q\left(s_{i,n},a_{i,n}\right)\right]$$

where $Q(s_{i,n},a_{i,n})$ denotes the current estimate of the expected cumulative reward for taking action $a_{i,n}$ in state $s_{i,n}$, while α represents the learning rate that determines how strongly new information influences previous estimates. The term $r_{i+1,n}$ corresponds to the immediate reward obtained after executing the action, and the discount factor γ regulates the relative importance of future rewards compared to immediate ones. The expression $\max_{a^{\prime }}Q\left(s_{i+1,n},a^{\prime }\right)$ captures the maximum expected return achievable from the next state $s_{i+1,n}$, computed by evaluating all possible actions and selecting the one with the highest Q-value stored in the Q-table. After each update, the refined value of $Q(s_{i,n},a_{i,n})$ is written back into the Q-table, enabling the agent to gradually improve its knowledge and converge toward an optimal control policy.

The state space *S* is defined using environmental and operational data collected from internal and external sensors installed in each home. Specifically, it includes:

- the time slot $t_{i}$,
- the day of the year $D_{i}$,
- the ambient weather condition $m_{i}$,
- the difference between indoor and outdoor temperatures $\left(T_{i,n}^{I}-T_{i}^{O}\right)$,
- the balance between electricity production $E_{i,n}^{p}$ and consumption $E_{i,n}^{c}$,
- the battery state of charge (SOC),
- the presence of occupants,
- the temperature of the domestic hot water tank $T_{i,n}^{P}$.

The action space *A* is defined by the controllable home automation devices available in each home:

- activation of the space heating/cooling system,
- activation of the water heating system,
- activation of the washing machine,
- activation of the dishwasher.

To ensure tractable learning and limit the dimensionality of the Q-table, both *S* and *A* were discretized. The number of discretization categories for each state variable was selected based on preliminary sensitivity analyses, with the objective of balancing control performance and convergence time, while avoiding an excessive combinatorial growth of the Q-table.

Several variables originally characterized by continuous or high-cardinality domains were reduced to a small number of representative levels. The time slot, simulated with 3 h increments (8 levels), was reduced to a binary distinction between day and night. The day of the year was mapped to three seasonal categories (winter, summer, mid-season), while weather conditions were reduced from five classes to a binary indicator of solar presence. Occupancy remained a Boolean variable.

Continuous variables were discretized using threshold-based rules. Indoor and outdoor temperatures were mapped to three levels depending on whether the indoor temperature was above, below, or within the comfort range, and whether the outdoor temperature differed by more than 2 °C. Electricity production and consumption were categorized into surplus, deficit, or balance. The battery SOC was discretized into two levels (above or below 50%), and the hot water temperature into three levels based on thresholds at 20 °C and 35 °C. This discretization results in 1296 possible states.

The action space was discretized similarly. The air conditioning control, ranging from −1 (cooling) to 1 (heating), was divided into nine equally spaced levels. The water heating command, ranging from 0 to 1, was discretized into five levels. The washing machine and dishwasher were modeled as Boolean actions. Overall, the action space comprises 180 possible actions.

Table 1 summarizes the discretization applied to each variable.

The static discretization of certain state variables, such as the time-slot indicator, was selected to provide a simplified yet functional representation of the environment for fast-training Q-learning. Although daylight duration varies seasonally, preliminary tests showed that a coarse discretization (e.g., day/night) is sufficient to demonstrate the learning algorithm’s effectiveness, while keeping computational complexity low. Future work could explore dynamic discretization or seasonal adaptation for even finer performance improvements.

### 2.3. Residential Environment

The Residential Environment represents the central component of the simulation framework, modeling the physical, behavioral, and operational characteristics of each dwelling within the REC. This block integrates external environmental conditions with internal household dynamics, combining sensor measurements, occupant behavior, and the response of home automation systems. By capturing the heterogeneity of building features, user habits, and controllable devices, the Residential Environment provides a realistic and dynamically evolving context in which the RL agent operates, ensuring that learning and decision-making occur under conditions representative of real residential settings.

#### 2.3.1. User Activity Profiles: Create a New Building

The generation of a new building follows a stochastic procedure that ensures diversity across the simulated residential stock while remaining consistent with real-world demographic and housing characteristics. Although the process relies on random sampling, the resulting household structures and dwelling properties are statistically aligned with national market analyses and demographic distributions reported by ISTAT and other authoritative sources [27,28,29,30]. This approach guarantees that the simulated homes reflect realistic variations in family composition, dwelling size, and occupancy patterns, thereby enhancing the credibility and representativeness of the model.

Beyond the structural and demographic aspects, the creation of a new building also involves defining the behavioral profiles that govern how occupants interact with the dwelling over time. These profiles determine presence patterns, appliance usage, and the timing of energy-intensive activities, all of which contribute to shaping the daily and seasonal dynamics of residential demand. By integrating probabilistic routines with temporal markers such as weekdays, weekends, holidays, and school breaks, the model captures the heterogeneity of real households and reproduces the variability that characterizes actual user behavior. This behavioral layer is essential for generating realistic load profiles and for providing the RL agent with a dynamic and non-deterministic environment in which to operate.

Once generated, each dwelling, together with its associated user activity profiles, is stored and subsequently reused throughout the simulation. This choice is essential for the reinforcement learning framework adopted in this work: each agent is trained on the specific characteristics of its corresponding home, learning to optimize energy use under the unique structural, behavioral, and environmental conditions of that dwelling. By grounding the training process in individualized residential contexts, the model supports the development of control strategies that are robust, adaptive, and capable of generalizing across diverse living environments.

#### 2.3.2. Building Energy Consumption and Thermal Behavior

Energy consumption is modeled at household level by coupling dwelling characteristics with occupant behavior. The number of occupants scales both the dwelling size and the intensity of end-uses, while thermal demand emerges from the interaction between indoor-outdoor temperatures, envelope properties, and system efficiency. This modeling choice ensures that the simulation captures both structural and behavioral variability, providing a realistic representation of residential energy dynamics.

**Figure 2:**
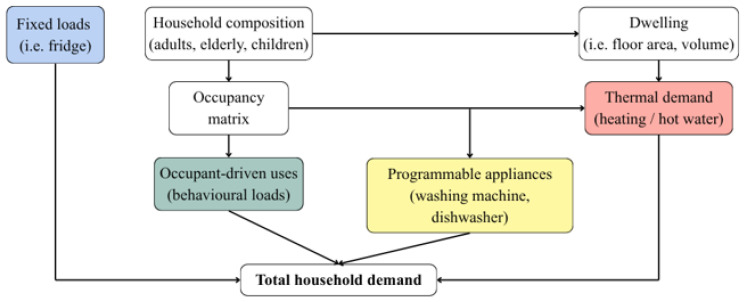
Logical flowchart of household energy demand components.

Figure 2 illustrates the logical flow of variables contributing to total household energy demand. The diagram highlights how different factors interact to shape the overall consumption profile.

- *Fixed loads* represent baseline consumption from non-controllable devices such as refrigerators, which operate continuously regardless of user presence.
- *Occupant-driven uses* include stochastic behavioral loads such as lighting and plug loads, which depend on user activity and presence.
- *Programmable appliances* such as washing machines and dishwashers, whose operation is scheduled by the agent based on user habits and optimization objectives.
- *Thermal demand* arises from the need to maintain indoor comfort through heating and hot water, influenced by envelope properties and external conditions.

Overall, the interaction between structural features, occupant behavior, and controllable loads provides a coherent representation of residential energy use. This decomposition clarifies the contribution of each component to total demand and supports a realistic modeling of household energy dynamics.

##### Fixed Loads and Occupant-Driven Uses

Fixed loads represent the portion of household electricity demand that remains largely independent of occupant behavior. These include appliances and systems that operate continuously or according to predefined cycles, such as refrigerators, standby electronics, network equipment, and essential lighting. Their contribution forms a stable baseline that persists across all time slots, ensuring that the dwelling maintains a minimum level of energy consumption even in the absence of occupants. In the simulation, these loads are modeled through predefined profiles that reflect typical residential patterns, capturing both the constancy and the slight variability associated with real-world appliance operation.

In contrast, occupant-driven uses introduce a dynamic and highly variable component to household energy demand. These loads arise from the presence and activities of household members and include personal device usage, small appliances, and behavior-dependent lighting. Their magnitude and timing depend on probabilistic presence profiles, daily routines, and contextual factors such as weekends, holidays, and school periods. A clear example of this variability is the additional consumption associated with meal preparation, which produces noticeable peaks during lunch and dinner time slots. In the model, these peaks are generated by scaling the cooking-related load according to the number of occupants effectively present in the dwelling at those times, thereby capturing one of the most characteristic behavioral signatures of residential electricity use. By combining individual consumption patterns with stochastic presence vectors, the model reproduces the temporal heterogeneity typical of real households and provides the RL agent with a realistic and evolving demand landscape.

##### Programmable Appliances

Programmable appliances, such as the washing machine and dryer, introduce an additional layer of complexity into the household energy model, as their operation depends not only on electrical availability but also on the production and storage of domestic hot water. In this simulator, these appliances are assumed to be modern, high-efficiency devices that require both electricity and hot water to complete their cycles. As a result, their activation influences not only the electrical load but also the thermal demand associated with water heating. This creates an implicit coupling between appliance scheduling and the home automation actions responsible for maintaining the hot water reserve temperature, meaning that the agent must coordinate multiple control variables to ensure that sufficient hot water is available when needed.

To preserve realistic usage patterns, the model also enforces behavioral constraints on appliance operation. The washing machine can be used at most twice per day, while the dryer is limited to a single daily cycle. Furthermore, an appliance cannot be restarted immediately after completing a cycle unless an occupant is present in one of the subsequent time slots to unload and reload it. This requirement reflects the natural dependency between user presence and appliance availability: even if energy conditions are favorable, the device remains locked until a household member performs the necessary manual interaction. By combining physical constraints, behavioral rules, and resource dependencies, the simulator provides a realistic representation of programmable appliance usage and offers the RL agent a rich, multi-dimensional decision space in which to operate.

##### Thermal Demand: Hot Water

The thermal demand for domestic hot water (DHW) is determined by the volumes extracted during sanitary uses and by the programmable appliances that require heated water. For each minute *t*, the thermal energy required to raise the extracted volume $V_{\mathrm{draw}}\left(t\right)$ from the tank temperature $T_{\mathrm{tank}}\left(t\right)$ to the delivery temperature $T_{\mathrm{draw}}\left(t\right)$ is computed as:

(3) $$Q_{\mathrm{draw}}\left(t\right)=V_{\mathrm{draw}}\left(t\right)\;c_{H_{2}O}\left[T_{\mathrm{draw}}\left(t\right)-T_{\mathrm{tank}}\left(t\right)\right].$$

The total DHW demand in minute *t* is the sum of all simultaneous draws:

(4) $$Q_{\mathrm{DHW}}\left(t\right)=\sum_{i=1}^{N_{\mathrm{draws}}}Q_{\mathrm{draw},i}\left(t\right).$$

The heat pump can supply thermal energy up to its maximum heating power $P_{\mathrm{HP},\max}$. The effective heat delivered in minute *t* is therefore:

(5) $$Q_{\mathrm{HP}}\left(t\right)=\min\left(Q_{\mathrm{DHW}}\left(t\right),\;P_{\mathrm{HP},\max}\cdot 60\right).$$

Whenever the thermal demand exceeds the heat pump capacity, the remaining portion must be supplied by the auxiliary booster:

(6) $$Q_{\mathrm{boost}}\left(t\right)=\max\left(0,\;Q_{\mathrm{DHW}}\left(t\right)-Q_{\mathrm{HP}}\left(t\right)\right).$$

The behavior of the auxiliary booster is summarized through the indicator $b_{i,n}$, introduced earlier in the mathematical formulation. This quantity expresses how frequently the system must rely on the booster to satisfy the domestic hot water demand when the heat pump alone is insufficient. In practice, $b_{i,n}$ increases whenever a draw event occurs under conditions in which the tank temperature is too low or the instantaneous thermal demand exceeds the heat pump capacity. Conversely, it remains close to zero when the agent maintains an adequate thermal reserve and schedules heating actions effectively. Since booster activation is significantly less efficient than heat pump operation, $b_{i,n}$ serves as a direct measure of thermal management quality and is therefore incorporated into the reward structure to penalize strategies that lead to frequent or unnecessary booster usage.

This metric quantifies how often the system relies on the auxiliary heater rather than the heat pump alone. A lower value indicates that the agent successfully maintained an adequate thermal reserve in the tank, avoiding inefficient booster interventions. Conversely, a high value reflects poor scheduling of heating actions or excessive simultaneous hot water usage.

The total thermal demand over a time slot is obtained by summing the minute-by-minute contributions:

(7) $$Q_{\mathrm{DHW},\mathrm{slot}}=\sum_{t=1}^{60}Q_{\mathrm{DHW}}\left(t\right).$$

By explicitly modeling the deficit between demand and heat pump capacity, and by tracking the frequency of booster activation, the simulator provides a detailed representation of DHW thermal dynamics. The resulting booster_fraction serves as a direct indicator of thermal management efficiency and is used in the reward function to encourage strategies that minimize auxiliary heating.

##### Thermal Demand: Heating

The thermal demand for space heating is evaluated minute by minute by simulating how the indoor temperature evolves under the combined effect of heat losses and the thermal power supplied by the heating system. At the beginning of the simulation, the indoor temperature is initialized within a realistic range to represent different initial thermal states of the dwelling. During each minute, the temperature decreases due to heat dispersion through the building envelope and increases when the heating system is active.

Heat losses depend on the temperature difference between indoor and outdoor air and on the thermal characteristics of the walls. This effect is expressed through a single temperature–loss relation:

(8) $$\Delta T_{\mathrm{loss}}=\frac{k_{\mathrm{walls}}\;A_{\mathrm{walls}}\;\left(T_{\mathrm{in}}-T_{\mathrm{out}}\right)\cdot 60}{C_{\mathrm{air}}}.$$

The parameters in Equation (8) are defined as follows: $k_{walls}$ is the thermal transmittance of the building envelope (W/m^2^K), $A_{walls}$ is the total wall area (m^2^), and $C_{air}$ represents the thermal capacitance of indoor air (J/K). Values for these parameters are obtained from typical building standards for residential houses at the considered location, based on architectural plans and material properties, ensuring realistic energy demand modeling for the simulation.

In parallel, the heating system provides thermal energy according to the power level selected by the home automation agent. The corresponding temperature increase is computed as:

(9) $$\Delta T_{\mathrm{heat}}=\frac{\eta_{\mathrm{heat}}\;P_{\mathrm{heat}}\cdot 60}{C_{\mathrm{air}}}.$$

The indoor temperature is then updated by combining the two contributions:

(10) $$T_{\mathrm{in}}=T_{\mathrm{in}}+\Delta T_{\mathrm{heat}}-\Delta T_{\mathrm{loss}}.$$

The electrical consumption associated with heating is obtained from the requested power and accumulated minute by minute over the entire time slot. The resulting value is added to the total household energy demand, providing a realistic representation of the dwelling’s thermal behavior and the impact of heating actions throughout the day.

#### 2.3.3. Building Energy Production

The photovoltaic energy production of each dwelling is computed as a function of three input variables: the day of the year $D_{i}$, the atmospheric condition $m_{i}$, and the time interval $t_{i}$. These inputs determine the position of the sun, the clearness of the sky, and the irradiance incident on the photovoltaic modules, as defined in [19].

For each day $D_{i}$, the solar declination $\delta (D_{i})$ is evaluated, and the corresponding irradiance reduction factor $\sigma (m_{i})$ is obtained from the weather–clearness map. The clearness index is then computed as

(11) $$k_{t}\left(D_{i},m_{i}\right)=0.177+0.692\;\sigma \left(m_{i}\right),$$

which modulates the clear-sky irradiance according to the observed weather condition.

For each time interval $t_{i}$, the solar zenith angle $\theta_{z}\left(D_{i},t_{i}\right)$ is evaluated, and the clear-sky global horizontal irradiance is computed through the Haurwitz model. The actual irradiance on the horizontal plane is then obtained as

(12) $$GHI_{\mathrm{real}}\left(D_{i},m_{i},t_{i}\right)=k_{t}\left(D_{i},m_{i}\right)\;GHI_{\mathrm{clear}}\left(D_{i},t_{i}\right).$$

The irradiance incident on the tilted photovoltaic surface is obtained by combining the direct, diffuse, and ground-reflected components according to the module tilt β and azimuth:

(13) $$I_{\mathrm{tilted}}\left(D_{i},m_{i},t_{i}\right)=DNI\cos\theta +DHI\frac{1+\cos\beta }{2}+GHI_{\mathrm{real}}\;\rho \;\frac{1-\cos\beta }{2},$$

where θ is the incidence angle, which depends on both the tilt β and the azimuth, and ρ is the ground reflectance.

The electrical energy produced during interval $t_{i}$ is then computed as

(14) $$E_{PV}\left(D_{i},m_{i},t_{i}\right)=P_{\mathrm{rated}}\;\frac{I_{\mathrm{tilted}}\left(D_{i},m_{i},t_{i}\right)}{G_{PV,std}},$$

with $P_{\mathrm{rated}}$ the nominal peak power of the PV array and $G_{PV,std}$ the standard irradiance (1000 W/m^2^). This formulation implicitly accounts for reduced performance at low irradiance levels through the irradiance–power scaling applied to each module.

This formulation provides a compact representation of the photovoltaic generation process while preserving the dependence on the key environmental and temporal variables that drive the energy production of each dwelling.

When applying the framework to a different geographical location, the photovoltaic production model can be recalibrated by replacing the irradiance and ambient temperature datasets and adjusting installation-specific parameters (e.g., panel efficiency and nominal power), thus ensuring consistency with local environmental conditions.

#### 2.3.4. Battery Storage and Energy Management

The battery is the core element that enables temporal shifting of energy within the household. Its behavior is entirely governed by the evolution of the state of charge (SOC), which determines whether the system can store surplus photovoltaic energy, supply energy during demand peaks, or remain idle, as defined in [19]. For each time slot, the SOC is updated according to the actual charging and discharging actions computed in the previous sections.

When photovoltaic generation exceeds household consumption, a portion of the surplus can be stored in the battery. The amount of energy actually stored in the current time slot, indicated as $E_{\mathrm{ch}}$, already incorporates the charging efficiency and respects the limits on maximum power and allowable SOC range. In this situation, the state of charge increases proportionally to the energy that the system is able to store.

In periods where household demand is higher than photovoltaic production, the battery provides energy to reduce the deficit. The discharged energy, denoted by $E_{\mathrm{dc}}$, accounts for discharge efficiency and is constrained by both the minimum SOC threshold and the maximum discharge power. As a result, the state of charge decreases according to the energy supplied to the household.

Through this mechanism, the SOC evolves over time as the direct outcome of all charging and discharging actions. Its trajectory reflects the battery’s ability to shift energy across different time slots, absorb renewable surplus, and mitigate consumption peaks. For the control agent, managing the SOC is therefore essential to maintain operational flexibility and ensure that sufficient storage capacity is available when needed.

The nominal battery capacity, indicated as $C_{\mathrm{Cap}}$, provides the scaling factor that links charged or discharged energy to the corresponding variation in SOC. The SOC therefore evolves as a discrete-time state variable that reflects the cumulative effect of all charging and discharging processes:

(15) $$SOC_{t+1}=SOC_{t}+\frac{E_{\mathrm{ch}}}{C_{\mathrm{Cap}}}-\frac{E_{\mathrm{dc}}}{C_{\mathrm{Cap}}}.$$

This formulation highlights the role of the battery as a flexible energy buffer. A high SOC increases the household’s ability to rely on stored energy during periods of low photovoltaic production, while a low SOC provides headroom for storing future renewable surplus. Effective energy management therefore requires the control agent to regulate the SOC over time, anticipating future conditions and balancing immediate needs with long-term flexibility. The SOC trajectory becomes a direct indicator of the agent’s ability to coordinate storage, consumption, and renewable generation within the household energy system.

## 3. Rewards

The evaluation of agent performance within the simulated REC relies on a reward system that integrates multiple dimensions of household and community behavior. Each house is equipped with sensors capable of monitoring key variables such as indoor temperature, energy production from photovoltaic panels, energy consumption from appliances, and the operational status of devices. These measurements provide the basis for computing the different reward components associated with comfort, economic efficiency, and sustainability.

The overall reward $r_{i,n}$ for the *n*-th agent at time step *i* is obtained as a weighted sum of three distinct contributions: Thermal and Service Comfort ($r_{i,n}^{c}$), Economic Saving ($r_{i,n}^{e}$), and Green Sustainability ($r_{i,n}^{g}$). Formally, this can be expressed as:

(16) $$r_{i,n}=\frac{w_{1}\cdot r_{i,n}^{c}+w_{2}\cdot r_{i,n}^{e}+w_{3}\cdot r_{i,n}^{g}}{w_{1}+w_{2}+w_{3}}$$

where $w_{1}$, $w_{2}$, and $w_{3}$ are weighting coefficients that allow the prioritization of different objectives. In the simulations presented in this work, all three weights have been set equal to 1, thereby giving equal importance to comfort, economic savings, and sustainability.

Although equal weighting coefficients are adopted in this study to provide a neutral baseline configuration, the proposed framework allows user-specific customization of the reward weights. This enables prosumers and consumers to prioritize comfort, economic savings, or sustainability differently, reflecting heterogeneous preferences within the REC.

The following subsections provide a detailed description of each reward component, outlining how sensor data and system variables are used to quantify the different aspects of household and community performance.

### 3.1. Thermal and Service Comfort

The Thermal and Service Comfort reward $r_{i,n}^{c}$ evaluates the indoor environment of each House by considering three main aspects: indoor temperature, the share of booster energy used, and the scheduling of major household appliances such as the washing machine and dishwasher. Its purpose is to incentivize actions that maintain acceptable comfort conditions while balancing energy use.

When at least one occupant is present in the dwelling ($O_{i,n}^{T}=1$), the reward depends on the indoor temperature $T_{i,n}^{I}$. The score is highest in the optimal comfort range (18–22 °C), lower in mildly acceptable ranges, and negative when the temperature is too cold or too hot. If no occupant is present ($O_{i,n}^{T}=0$), the reward is set to zero:

(17) $$r_{i,n}^{\mathrm{temp}}=\left\{\begin{array}{cc} +1 & \mathrm{if}\;18<T_{i,n}^{I}\leq 22\\ +0.5 & \mathrm{if}\;15<T_{i,n}^{I}\leq 18\;\mathrm{or}\;22<T_{i,n}^{I}\leq 25\\ -0.5 & \mathrm{if}\;10<T_{i,n}^{I}\leq 15\;\mathrm{or}\;25<T_{i,n}^{I}\leq 30\\ -1 & \mathrm{if}\;T_{i,n}^{I}\leq 10\;\mathrm{or}\;T_{i,n}^{I}>30\\ 0 & \mathrm{if}\;O_{i,n}^{T}=0 \end{array}\right.$$

In this framework, hot water is primarily supplied by a heat pump, which is activated by the agent and provides efficient heating. The fraction of booster usage is represented by the variable $b_{i,n}$, which directly influences the comfort reward. From the user’s perspective, the need to activate the booster reflects a temporary lack of hot water availability, resulting in waiting time before services such as showers can be used; this delay negatively affects perceived comfort. The booster reward depends on the fraction of auxiliary energy used $b_{i,n}$:

(18) $$r_{i,n}^{\mathrm{booster}}=\left\{\begin{array}{cc} +2 & \mathrm{if}\;b_{i,n}\leq 0.25\\ +1 & \mathrm{if}\;0.25<b_{i,n}\leq 0.50\\ -1 & \mathrm{if}\;0.50<b_{i,n}\leq 0.75\\ -2 & \mathrm{if}\;b_{i,n}>0.75 \end{array}\right.$$

The evaluation of appliance usage within the comfort reward is based on the daily operation of the washing machine and dishwasher. Each household is constrained to a maximum of two washing machine cycles and one dishwasher cycle per day. For this reason, the reward must be considered across the eight time slots ($t_{i}$) that compose a full day.

For the washing machine, each activation within the allowed limit yields a bonus of +7, while each time slot in which the appliance is not used contributes a penalty of −1. If the agent activates the washing machine twice in a day, the daily reward results from two positive contributions and six negative ones, leading to a total of +8. This corresponds to an average reward of +1 per time slot. Conversely, if the washing machine is never activated, the daily penalty amounts to −8, with an average reward of −1 per time slot. The reward function is defined as:

(19) $$r_{i,n}^{\mathrm{washing}\;\mathrm{machine}}=\left\{\begin{array}{cc} +7 & \mathrm{if}\;\mathrm{washing}\;\mathrm{machine}\;\mathrm{is}\;\mathrm{used}\\ -1 & \mathrm{otherwise} \end{array}\right.$$

For the dishwasher, the logic is analogous. A single activation yields a bonus of +15, while the remaining seven time slots contribute penalties of −1. The daily balance is therefore +8, corresponding to an average reward of +1 per time slot. If the dishwasher is never activated, the daily penalty amounts to −8, again with an average reward of −1 per time slot. The reward function is expressed as:

(20) $$r_{i,n}^{\mathrm{dishwasher}}=\left\{\begin{array}{cc} +15 & \mathrm{if}\;\mathrm{dishwasher}\;\mathrm{is}\;\mathrm{used}\\ -1 & \mathrm{otherwise} \end{array}\right.$$

This formulation ensures that the agent is incentivized to schedule appliance usage appropriately within the daily cycle, balancing comfort and service provision with energy efficiency. By distributing the evaluation across the eight time slots, the reward structure captures both the necessity of appliance operation and the penalties associated with insufficient use.

A negative reward is assigned when a declared flexible appliance is not activated within the daily scheduling window, as this represents a missed opportunity to contribute to the coordinated energy balancing strategy of the REC and may require compensation from other members to maintain optimal collective performance.

The overall comfort reward for the *n*-th house at time step *i* is computed as the sum of the three components:

(21) $$r_{i,n}^{c}=r_{i,n}^{\mathrm{temp}}+r_{i,n}^{\mathrm{booster}}+r_{i,n}^{\mathrm{washing}\;\mathrm{machine}}+r_{i,n}^{\mathrm{dishwasher}}$$

Unlike the other reward components in the community framework, the comfort reward is not aggregated at the community level. Instead, it remains specific to each household, reflecting the inherently individual nature of thermal comfort and appliance usage. This formulation ensures that each dwelling optimizes its own comfort conditions independently, while still contributing to the broader optimization of energy management within the REC.

### 3.2. Economic Saving

The Economic Saving reward $r_{i,n}^{e}$ measures the financial profitability of each household, considering both scenarios with and without energy sharing among community members. The objective is to encourage energy-efficient and cooperative strategies that maximize overall benefits at the REC level.

By encouraging self-consumption and reducing energy exchanges with the external grid, the economic reward component indirectly mitigates potential congestion effects in the distribution network, even though explicit congestion penalties are not currently modeled.

The first step is to define the surplus and deficit energy of each household:

(22) $$E_{i,n}^{p,ex}=\max\left(0,\;E_{i,n}^{p}-E_{i,n}^{c}\right),\;E_{i,n}^{c,ex}=\max\left(0,\;E_{i,n}^{c}-E_{i,n}^{p}\right)$$

Here, $E_{i,n}^{p,ex}$ represents the surplus energy produced by the household beyond its own demand, while $E_{i,n}^{c,ex}$ denotes the energy demanded in excess of its local production.

Based on these quantities, the proportional factor $I_{p}$ is defined as:

(23) $$I_{p}=\frac{E_{i,n}^{p,ex}}{\sum_{n=1}^{N}E_{i,n}^{p,ex}}+\frac{E_{i,n}^{c,ex}}{\sum_{n=1}^{N}E_{i,n}^{c,ex}}$$

with *N* being the number of REC households. The factor $I_{p}$ increases when a household either contributes a significant portion of the total community surplus or consumes a large share of the community’s overall deficit. This formulation rewards both efficient producers and active consumers, fostering fair and cooperative participation in the shared energy exchange process.

The profit without sharing energy in REC, as the user does not participate in a REC, is expressed as:

(24) $$\Pi_{i,n}^{\mathrm{noREC}}=E_{i,n}^{p,ex}\cdot c_{v}-E_{i,n}^{c,ex}\cdot c_{a}$$

where $c_{v}$ and $c_{a}$ denote the selling and purchase prices of electricity.

When energy sharing is allowed within the REC, the profit becomes:

(25) $$\Pi_{i,n}^{\mathrm{REC}}=E_{i,n}^{p,ex}\cdot c_{v}+I_{p}\cdot E_{i,n}^{\mathrm{sh}}\cdot \frac{I}{2}-E_{i,n}^{c,ex}\cdot c_{a}$$

where $E_{i,n}^{\mathrm{sh}}$ is the energy exchanged inside the community and *I* is the incentive for shared energy.

The relative gain between the shared and non-shared scenarios is defined as:

(26) $$R_{i,n}^{\mathrm{GE}}=\left\{\begin{array}{cc} \frac{\Pi_{i,n}^{\mathrm{REC}}}{\Pi_{i,n}^{\mathrm{noREC}}}, & \Pi_{i,n}^{\mathrm{REC}},\Pi_{i,n}^{\mathrm{noREC}}>0\\ \frac{\Pi_{i,n}^{\mathrm{noREC}}}{\Pi_{i,n}^{\mathrm{REC}}}, & \Pi_{i,n}^{\mathrm{REC}},\Pi_{i,n}^{\mathrm{noREC}}<0 \end{array}\right.$$

The resulting economic score is mapped through a logarithmic transformation and clipped to the interval [−4,4]:

(27) $$r_{i,n}^{e}=k\cdot \log_{10}\left(R_{i,n}^{\mathrm{GE}}\right)-4$$

where *k* is a constant, set equal to 80/3 in these simulations.

Finally, to ensure robustness and fairness among all agents, the community-level economic reward is computed as:

(28) $$r_{i}^{e}=\frac{\frac{\sum_{n=1}^{N}r_{i,n}^{e}}{N}+\min\left(r_{i,n}^{e}\right)}{2}$$

Table 2 summarizes the decision logic underlying the economic reward. The structure shows how the reward is assigned depending on the sign of profits in both scenarios, with and without energy sharing. When both profits are positive, the reward is scaled logarithmically to capture relative gains, while mismatched signs are clipped to fixed values to ensure stability. This representation provides a concise overview of the reward mechanism, highlighting its role in promoting fairness and cooperative efficiency within the REC.

### 3.3. Green Sustainability

The Green Sustainability reward $r_{i,n}^{g}$ evaluates the share of renewable energy utilized within the community, penalizing dependence on fossil-based electricity. It integrates local self-consumption, intra-community sharing, and interactions with the external grid.

At each time step, the renewable and fossil contributions are computed as:

(29) $$E_{i,n}^{\mathrm{rinn}}=\max\left(0,\;E_{i,n}^{p}-E_{i,n}^{c}\right)+E_{i,n}^{\mathrm{sh},\mathrm{rinn}},\;E_{i,n}^{\mathrm{foss}}=E_{i,n}^{c}-E_{i,n}^{\mathrm{rinn}}$$

where $E_{i,n}^{\mathrm{sh},\mathrm{rinn}}$ denotes the portion of renewable energy redistributed within the community. In practice, this redistribution is computed iteratively to ensure equitable allocation among households with energy deficits, until the available shared energy is fully consumed. This mechanism models a fair and cooperative internal balancing of renewable energy use before any interaction with the external grid.

The green ratio is then defined as:

(30) $$R_{i,n}^{\mathrm{green}}=\frac{E_{i,n}^{\mathrm{rinn}}+\alpha }{E_{i,n}^{\mathrm{rinn}}+E_{i,n}^{\mathrm{foss}}+\alpha }$$

where α is a small regularization term preventing division by zero (in this work, it is set as α=0.68). This indicator ranges in [0,1], from fully fossil-based consumption ($R_{i,n}^{\mathrm{green}}\approx 0$) to completely renewable use ($R_{i,n}^{\mathrm{green}}=1$).

To provide a stronger incentive toward high-renewable scenarios, a non-linear transformation is applied:

(31) $$r_{i,n}^{g}=\frac{8}{9}\cdot 10^{R_{i,n}^{\mathrm{green}}}-\frac{44}{9}$$

which amplifies marginal improvements when the share of renewable energy approaches unity, thus favoring sustainable and self-sufficient operation modes. The resulting reward is bounded within the range [−4,4] and scaled to ensure consistency with the economic and comfort components.

Finally, to promote both fairness and collective sustainability, the community-level green reward is computed as:

(32) $$r_{i}^{g}=\frac{\frac{\sum_{n=1}^{N}r_{i,n}^{g}}{N}+\min\left(r_{i,n}^{g}\right)}{2}$$

This averaging strategy discourages unbalanced configurations where a few agents dominate renewable usage while others rely on fossil energy, encouraging a cooperative equilibrium across the community.

## 4. Results

This section presents the results obtained from the proposed reinforcement learning framework applied to a small-scale Renewable Energy Community (REC). The reference REC consists of eleven heterogeneous households, of which five operate as *prosumers*, equipped with photovoltaic generation and battery storage, and six operate as *consumers*, relying solely on energy demand management. These households differ in demographic composition and consumption patterns, providing a realistic and diverse testbed for evaluating the learning and coordination capabilities of the proposed approach.

The analysis is structured into two main parts. First, each household is trained individually through a dedicated pre-training phase, where the agent learns to manage its local energy system while interacting with a virtual representation of the remaining community. This procedure allows the agent to explore the state–action space efficiently and to acquire a stable and well-defined policy before being deployed in the multi-agent environment. The benefits of this approach are quantified through several performance evaluation of reward.

In the second part, the pre-trained agents are integrated into the full REC simulation. Here, the objective is to assess whether the policies learned during isolated training remain stable when the agents operate simultaneously and interact through shared energy exchanges. A stable reward profile in this phase indicates that the pre-training procedure successfully equips each agent with a robust and transferable policy, capable of generalizing to the multi-agent setting without degradation.

The motivation for adopting this two-stage training strategy lies in the intrinsic instability of multi-agent reinforcement learning under ε-greedy exploration. When multiple agents explore simultaneously, the environment becomes highly non-stationary, often leading to slow convergence or failure to learn meaningful policies. By contrast, the proposed pre-training approach is computationally efficient, scalable, and well suited for distributed implementations: each household device can autonomously train its own agent using a simulated community model, and subsequently validate the learned policy within the real REC environment.

The following sections present the detailed results of this process, beginning with the single-house pre-training outcomes and followed by the performance of the complete energy community. The parameters used for simulations are reported in Table 3.

### 4.1. Single House Pre-Training Results

**Figure 3:**
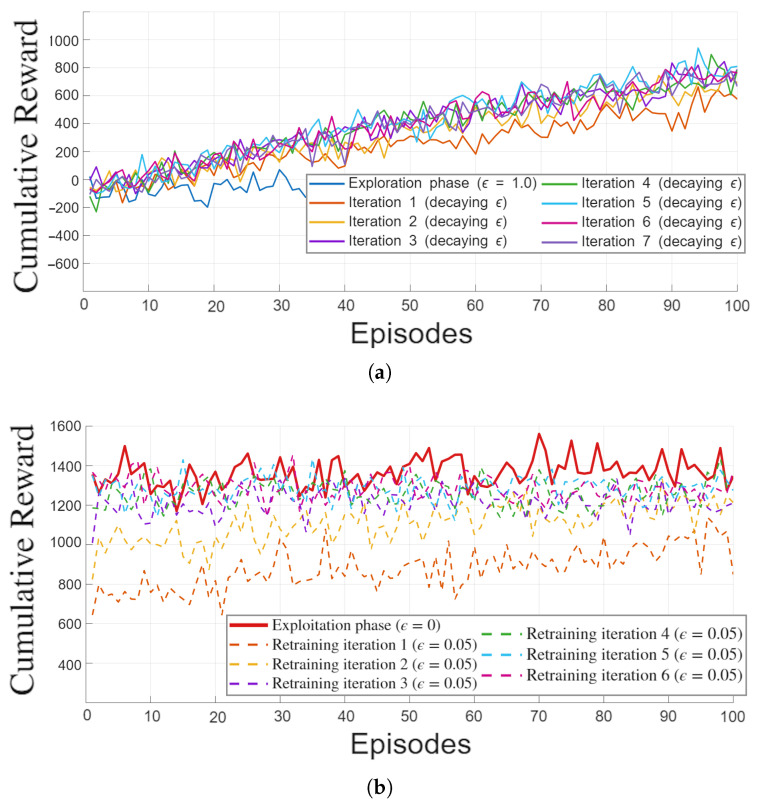
Cumulative reward trajectories for a single-house agent, illustrating the transition from ε-decaying training (**a**) to low-exploration retraining and full exploitation (**b**). Figure (**a**) is an example of cumulative reward trend for single house during the training phase. Figure (**b**) is an example of cumulative reward trend for single house during the retraining and exploitation phases.

The first part of the analysis focuses on the pre-training of individual households, carried out using a single-house virtual model that embeds the aggregated energy flows of the remaining dwellings in the community. The Renewable Energy Community considered in this study consists of eleven buildings derived from the same five representative family types. Five of these dwellings operate as prosumers, while the remaining six act as consumers and share the same demographic structures but differ in their behavioral patterns. Despite these differences, the learning dynamics observed during pre-training are qualitatively consistent across all agents. For this reason, a single representative example is reported in Figure 3.

Figure 3a illustrates the cumulative reward evolution during the ε-decaying training phase. The agent initially operates under a fully exploratory regime, which results in low and highly variable rewards. As the value of ε decreases linearly across iterations, the agent progressively shifts from random exploration to exploitation of the most promising actions. This transition produces a clear and monotonic improvement in the cumulative reward, with successive iterations becoming smoother and more stable. Convergence is reached when the variation between the last two iterations falls below the predefined threshold of approximately five percent, indicating that further exploration no longer yields meaningful improvements.

Once convergence is achieved, the model undergoes an additional refinement stage, shown in Figure 3b. In this phase, the agent is retrained with a small but constant exploration rate, which allows the policy to be consolidated under quasi-deterministic conditions. Across these iterations, the cumulative reward continues to increase while the variability across episodes decreases, confirming the stability of the learned behavior. Finally, in the pure exploitation phase, the agent consistently achieves the highest reward levels, demonstrating that the policy obtained during pre-training is both robust and near-optimal.

The total training time of the proposed RL agent is approximately 2 h. This corresponds to 8 independent training runs, each lasting about 20 min and simulating 100 years of operation. The simulator was implemented and executed on Google Colab, and the reported times refer to this computational environment. Although the overall training time is not excessive, further optimizations are possible. In particular, improvements may include reducing the number of training runs, tuning the training duration, and optimizing the code implementation. At present, the framework does not exploit parallel execution, which represents an additional opportunity for reducing convergence time in future developments.

Overall, the results confirm the effectiveness of the single-house pre-training strategy. By isolating each agent during the learning process, the method avoids the non-stationarity and slow convergence typically associated with multi-agent ε-greedy exploration. The resulting policies are stable, efficient, and well suited for deployment in the full energy community, where their performance will be further assessed in the subsequent REC-level simulations.

#### Performance Evaluation

**Figure 4:**
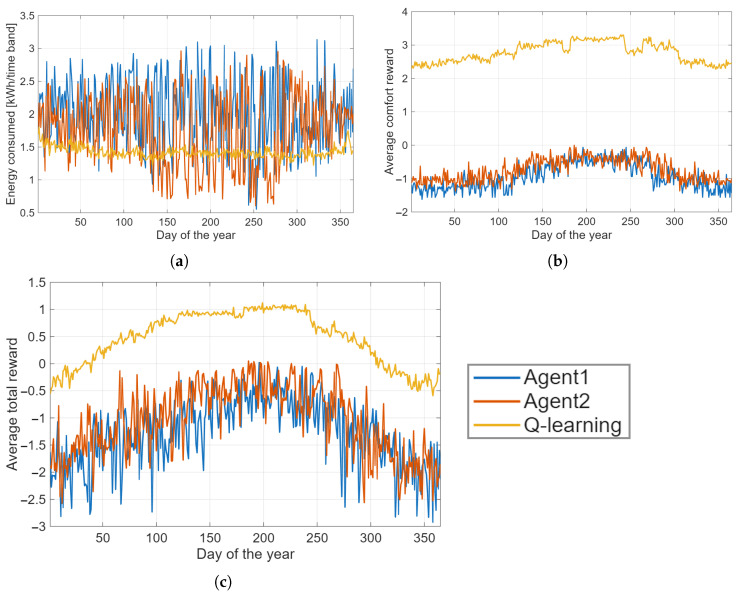
Performance comparison between the three controllers, where Agent1 and Agent2 represent two baseline rule-based strategies and the third controller is based on Q-learning, showing average daily energy consumption (**a**), comfort reward (**b**), and overall reward (**c**) computed over a full year of simulation. Figure (**a**) shows the average daily energy consumption for the three different control strategies. Figure (**b**) shows the average daily comfort reward for the three different control strategies. Figure (**c**) shows the average daily total reward for the three different control strategies.

To provide a compact and comparative view of the three control strategies, Figure 4 summarizes their average daily performance over an entire year of simulation. The figure reports three complementary indicators: energy consumption in Figure 4a, comfort reward in Figure 4b, and the resulting total reward in Figure 4c. Together, these metrics offer a comprehensive assessment of how each controller balances thermal comfort, energy efficiency, and overall operational quality.

Agent1 and Agent2 are rule-based baseline strategies designed to provide reference points for evaluating the Q-learning approach. Agent1 uses simple time-based heuristics: it discretizes presence, time slot, and temperature difference (ΔT) and controls appliances accordingly. For example, the washing machine is activated only during daytime with a limit of one usage per day, the dishwasher is used only at night, and the heat pump/climate control is adjusted based on indoor-outdoor temperature differences. Agent2 follows a more energy-aware heuristic: it considers residual energy in the system to anticipate appliance operation, allowing the washing machine and dishwasher to run earlier if excess renewable energy is available, while also adjusting climate control according to temperature and energy availability. Both strategies do not learn from the environment but follow deterministic rules, providing a baseline against which the adaptive and cooperative performance of the Q-learning agent can be compared.

Figure 4a shows that the Q-learning agent achieves the lowest and most stable energy consumption among the three strategies. While the two rule-based controllers exhibit higher demand and larger fluctuations, particularly during transitional seasons, the learned policy consistently aligns heating and appliance operation with renewable availability, thereby reducing grid withdrawals and improving self-consumption.

The comfort-related performance, illustrated in Figure 4b, further highlights the advantages of the reinforcement learning approach. The Q-learning agent maintains indoor conditions close to the comfort band with significantly lower variance, whereas the deterministic controllers tend to oscillate more widely due to their fixed thresholds and limited adaptability to external conditions. This stability reflects the agent’s ability to anticipate thermal dynamics and adjust its actions proactively.

Finally, the aggregated total reward in Figure 4c confirms the superior overall performance of the Q-learning controller. Its positive average reward indicates a well-balanced trade-off between comfort preservation and energy efficiency, whereas both rule-based agents yield negative values, revealing their difficulty in simultaneously satisfying the multiple objectives embedded in the reward structure.

Overall, the results presented in Figure 4 demonstrate that the policy learned during the single-house pre-training phase generalizes effectively when deployed on the physical model. The Q-learning agent consistently outperforms the deterministic baselines across all metrics, combining comfort stability, reduced energy consumption, and improved global reward. These findings validate the robustness of the pre-training strategy and confirm the suitability of reinforcement learning for residential energy management in dynamic and uncertain environments.

Table 4 provides a quantitative summary of the main performance indicators for all controllers. The table complements the graphical results by enabling a direct comparison of absolute and relative improvements, highlighting the superior stability and efficiency of the Q-learning agent relative to the deterministic baselines.

### 4.2. Renewable Energy Community Results

Building upon the insights gained from the single-house pre-training phase, this section presents the results of the full-scale deployment of the proposed learning framework within the Renewable Energy Community.

All eleven agents operate simultaneously within a shared environment, exchanging energy and adapting their decisions according to the collective reward structure. The goal is to evaluate the stability, scalability, and adaptability of the pre-trained policies when exposed to multi-agent interactions and realistic community dynamics.

**Figure 5:**
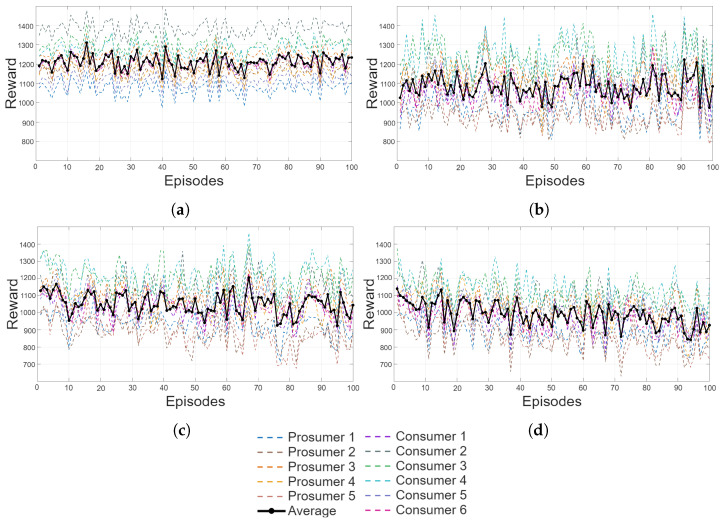
Community cumulative reward. (**a**) Static deployment (ε=0, no training): agents use the pre-trained policies without further updates. (**b**) Training without exploration (ε=0, with training): agents continue learning, but act deterministically without exploration. (**c**) Mild exploration (ε=0.01, with training): all agents adapt concurrently with limited stochastic exploration. (**d**) Stronger exploration (ε=0.05, with training): agents learn and explore more widely to test stability and convergence limits.

Figure 5 provides an overview of the community cumulative reward under the four learning configurations considered in this study. Each panel corresponds to a different exploration and training setup, allowing a direct comparison of the resulting collective behavior.

Figure 5a illustrates the static deployment scenario, where all agents operate deterministically using the pre-trained Q-tables without further updates. The reward trajectory is smooth and stable, confirming that the policies learned during the single-house phase generalize effectively when deployed concurrently in the community. This configuration serves as a baseline for assessing the impact of additional training or exploration.

Figure 5b shows the case in which agents continue updating their Q-tables while acting deterministically. Although no exploration is introduced, the ongoing training produces moderate oscillations in the cumulative reward, reflecting the adjustments made by each agent as it refines its policy in response to the shared environment. Despite these fluctuations, the system maintains a stable long-term trend, demonstrating that deterministic retraining does not compromise community-level coordination.

Figure 5c reports the behavior under mild exploration. With a small exploration rate, agents occasionally test alternative actions, enabling incremental policy improvements while preserving overall stability. The cumulative reward exhibits slightly higher variability compared to the deterministic cases, yet convergence remains consistent, indicating that limited exploration can be safely integrated into the community without destabilizing the collective dynamics.

Finally, Figure 5d presents the results obtained with a stronger exploration rate. In this configuration, the increased stochasticity leads to more pronounced oscillations and a lower mean reward. Although the system ultimately converges, the higher variability confirms that excessive exploration can temporarily disrupt the coordinated behavior established during pre-training.

Overall, the results in Figure 5 demonstrate that the pre-trained Q-learning policies retain strong generalization capabilities when deployed in a multi-agent community. Deterministic operation ensures the highest stability, while mild exploration supports adaptability without compromising convergence. These findings validate the robustness of the proposed learning framework and highlight its suitability for real-world REC applications, where coordinated and reliable operation is essential.

## 5. Discussion

The results presented in Section 4.1 highlight the effectiveness of the proposed pre-training strategy for single-house agents. Both the initial exploration phase (Figure 3a) and the subsequent refinement stage (Figure 3b) required only a few hours of computation per dwelling when executed on Google Colab, suggesting that the procedure is computationally feasible even if distributed across individual households. This makes the approach suitable for re-training whenever local conditions change, such as the installation of new generation systems or shifts in user behavior. While the refinement phase is commonly adopted in the literature, the extended pre-training with seven iterations of 100 episodes and a linearly decreasing ε may appear excessive. However, preliminary tests showed that shorter schedules with standard ε decay produced similar reward levels but resulted in significantly less stable behavior once the agent was deployed within the REC (Section 4.2). This suggests that the optimization landscape contains multiple near-optimal yet differently stable configurations, and that repeated exploration helps the agent converge toward a more robust optimum.

While direct comparison with existing optimized RL frameworks is challenging due to differences in problem formulation and agent interaction assumptions, the performance trends observed in our experiments align with reported results in the literature, demonstrating that the proposed Q-learning approach achieves competitive efficiency in energy optimization, comfort maintenance, and sustainability metrics.

The decentralized architecture significantly reduces computational burden by distributing the learning process across individual households. Each agent operates using locally available IoT data, and the training procedure can be executed offline prior to deployment, ensuring scalability even in large-scale RECs.

The performance evaluation in Section 4.1 further demonstrates the benefits of reinforcement learning at the household level. The RL agent consistently maintains indoor thermal comfort, ensures the availability of domestic hot water, and schedules appliance usage appropriately throughout the day. These improvements are clearly reflected in the comfort reward (Figure 4b), which represents the most tangible benefit for end users. Moreover, the RL controller achieves higher overall reward values (Figure 4c) while reducing average energy consumption compared to rule-based baselines (Figure 4a). These results confirm that the agent not only enhances user comfort but also improves energy efficiency, demonstrating the potential of data-driven control strategies for residential energy management.

The proposed decentralized approach inherently enhances privacy protection, as each household agent processes only locally generated IoT data (e.g., occupancy, temperature, consumption), and no raw sensor information is exchanged. Only aggregated reward-related information is shared, preventing disclosure of sensitive behavioral patterns.

Finally, the multi-agent experiments in Section 4.2 provide insight into how the pre-trained agents behave when integrated into a full Renewable Energy Community. A slight reduction in average reward is observed when agents interact with one another (Figure 5a) compared to their isolated performance (Figure 3b), reflecting the additional constraints and interdependencies introduced at the community level. Nevertheless, the system remains stable, and allowing agents to continue training without exploration (Figure 5b) leads to consistent reward trajectories. In contrast, introducing exploration during REC-level training (Figure 5c,d) results in increased variability and a gradual decline in performance. This behavior is expected, as two of the three reward components are community-based: a single agent taking random exploratory actions can negatively affect the collective outcome. These findings reinforce the importance of thorough pre-training and controlled exploration when deploying reinforcement learning in cooperative energy communities.

The tabular Q-learning approach is inherently limited by the size of the state–action space. In this study, a 1296×180 discrete representation was used, which remains manageable for the proposed fast-training methodology. For larger or more complex RECs, the state space would grow combinatorially, motivating future exploration of deep reinforcement learning methods, such as Deep Q-Networks or Actor–Critic architectures, capable of handling continuous and high-dimensional state representations.

## 6. Conclusions and Future Directions

This work investigated the application of reinforcement learning techniques to the management of Renewable Energy Communities, with a focus on the development of scalable and cooperative control strategies for residential energy systems. Starting from a single-house learning framework and extending it to a community of eleven heterogeneous dwellings, the study demonstrated that classical Q-learning can be effectively adapted to dynamic, multi-agent energy environments.

The results highlight three main findings. First, single-house pre-training plays a crucial role in ensuring stability and coordination once agents operate concurrently within the community. Second, cross-transfer learning between households with similar characteristics accelerates convergence without compromising performance, suggesting that pre-trained models can be reused efficiently across comparable dwellings. Third, in the full multi-agent configuration, the level of exploration strongly influences collective behavior: deterministic or low-exploration settings lead to stable and high-performing operation, whereas higher exploration rates introduce noise and temporarily reduce global reward.

Overall, the proposed framework demonstrates functionality, computational efficiency, and the ability to support cooperative energy management in a realistic REC scenario. Despite its simplicity, the Q-learning approach exhibits strong generalization capabilities and provides a solid foundation for more advanced learning architectures. Future research will focus on optimizing the training process, improving computational efficiency through code optimization and potential parallelization, and expanding the state–action space to enable transitions toward Deep Q-Learning or other function approximation methods, thereby enhancing performance and robustness.

Further developments will aim to increase the realism of the simulation environment by refining consumption and production models, incorporating smoother meteorological dynamics, additional renewable sources, community-level storage systems, and battery degradation models [31] accounting for capacity fade and efficiency losses over time. Explicit fairness metrics, such as equity-based reward normalization or variance-based benefit distribution indicators, will also be integrated to ensure balanced value sharing among REC participants.

Finally, future work will include systematic benchmarking of the proposed framework against alternative control strategies, such as rule-based methods, model predictive control, supervised learning models trained on offline-generated optimal schedules, and advanced reinforcement learning architectures [32], including Deep Q-Networks, Actor–Critic models, and multi-agent deep RL frameworks. These evaluations will provide quantitative assessments of performance, scalability, and trade-offs between model complexity, computational cost, and control effectiveness, supporting the design of efficient and generalizable solutions for real-world energy communities.

In summary, the study provides evidence that reinforcement learning can serve as a promising tool for the intelligent management of decentralized energy systems, paving the way for future research on adaptive and cooperative control in Renewable Energy Communities.

## Figures and Tables

**Table 1:** Discretization of state and action space variables.

**State Space *S* Variable**	**Original Domain**	**Discretized Levels**
Time slot $t_{i}$	8 levels (3 h steps)	2 (day/night)
Day of year $D_{i}$	365 days	3 (winter/summer/mid-season)
Weather condition $m_{i}$	5 classes	2 (sun/no sun)
Occupancy	Boolean	Boolean
Indoor/outdoor temperature	Continuous	3 (comfort-based)
Energy production/consumption	Continuous	3 (surplus/balance/deficit)
Battery SOC	Continuous	2 (above/below 50%)
Hot water temperature	Continuous	3 (20°–35° thresholds)
**Action Space A Variable**	**Original Domain**	**Discretized Levels**
Air conditioning action	[−1, 1]	9 levels
Water heating action	[0, 1]	5 levels
Washing machine	Boolean	Boolean
Dishwasher	Boolean	Boolean

**Table 2:** Economic reward policy by profit signs in REC vs. non-REC scenarios.

	Profit noREC >0	Profit noREC <0
**Profit REC >0**	$k\;\log_{10}\;\left(R_{i,n}^{\mathrm{GE}}\right)-4$	+4
**Profit REC <0**	−4	$k\;\log_{10}\;\left(R_{i,n}^{\mathrm{GE}}\right)-4$

**Table 3:** Simulation parameters.

Parameter	Value
Time step $t_{i}$	3 h (8 per day)
Episode duration	1 year
Training duration	100 years
Climate data	Perugia, Italy
REC size	11 dwellings (5 prosumers, 6 consumers)
PV panels per prosumer	16
Battery capacity	9.6 kWh
Battery charge efficiency $E_{\mathrm{ch}}$	0.95
Battery discharge efficiency $E_{\mathrm{dc}}$	0.95
Maximum charging power	4.8 kW
Discount factor γ	0.9
Learning rate α	0.01
Q-table size	1296 states × 180 actions

**Table 4:** Quantitative performance comparison of the three control strategies over a full year of simulation. Values indicate mean (μ) and standard deviation (σ) across 365 days.

Metric	Agent 1	Agent 2	Q-Learning
Total reward	−1.282±0.487	−1.058±0.487	0.467±0.487
Comfort reward	−0.935±0.298	−0.745±0.298	2.780±0.298
Daily energy consumption [kWh]	2.024±0.085	1.748±0.085	1.423±0.085

## Data Availability

The raw data supporting the conclusions of this article will be made available by the authors on request.
